# “Trust my morphology”, the key message from a kidney stone

**DOI:** 10.1007/s00240-021-01280-4

**Published:** 2021-07-23

**Authors:** Agnieszka Pozdzik, Carl Van Haute, Naim Maalouf, Emmanuel Letavernier, James C. Williams, Khashayar Sakhaee

**Affiliations:** 1 Nephrology and Dialysis Department, Kidney Stone Clinic, Centre Hospitalier Universitaire, Brugmann Hospital, Brussels, Belgium; 2 Faculty of Medicine, Université Libre de Bruxelles (ULB), Brussels, Belgium; 3 Department of Urology, KU Leuven, Katholieke Universiteit Leuven, University Hospitals Leuven, Leuven, Belgium; 4 Department of Internal Medicine, Division of Mineral Metabolism, Charles and Jane Pak Center for Mineral Metabolism and Clinical Research, UT Southwestern Medical Center, Dallas, TX USA; 5 Service des Explorations Fonctionnelles Multidisciplinaires, Tenon Hospital, AP_HP, Paris, France; 6 INSERM UMRS 1155, Université Pierre et Marie Curie-Paris VI-Sorbonne Universités, Paris, France; 7 Department of Anatomy, Cell Biology and Physiology, Indiana University School of Medicine, Indianapolis, IN USA

Dear Editor,

The Consensus Conference Group recently published an article in *Urolithiasis* highlighting the importance of urine and stone analysis in the metabolic evaluation of kidney stone formers (KSF) [1]. Indeed, each kidney stone contains the imprints of the conditions which created them during their ‘lifetime in the kidney’.

The morpho-constitutional classification method (MCC) established by Prof Michel Daudon correlates the morphological characteristics of stones with specific metabolic disorders [2]. Briefly, it distinguishes 7 types and 21 subtypes according to the crystalline composition and shape, color and structure of stones identified using an optical stereo-microscope (Table 1). This very specific method is easy to learn and provides the opportunity to quickly identify highly recurrent diseases, sometimes serious in their clinical consequences. Indeed, subtypes Ic, Ie, IIId, IVa2 and V are pathognomonic of specific clinical entities corresponding to primary hyperoxaluria type 1, enteric hyperoxaluria, hyperuricosuria with diarrhea, distal tubular acidosis and cystinuria, respectively. In 2019, the International COllaborative NEtwork on Kidney sTones (ICONEKT) encouraged medical community to integrate this method for rapid identification of disorders responsible for the highly recurrent diseases [3]. Despite growing evidence of the robust diagnostic value of Daudon’s MCC and its benefits in drawing up individualized treatment plans, this simple tool unfortunately still remains underexplored. Given the increasing prevalence of KSF and the worrying rise in cases in groups previously considered to be at lower risk of stones (for example, women and children), the aim of our “call to action letter” is to support the position of accurate stone analysis elegantly highlighted by the Consensus Conference that you recently published [1]. In this way, we hope to raise interest and encourage more physicians to consider Daudon’s MCC as a priority in their clinical practice in order to improve the care of KSFs.

**Table 1:** Daudon’s morpho-constitutional classification of kidney stones, main characteristics and corresponding etiologies

Type/subtype	Main component	Etiological orientation
Ia	Whewellite	Dietary hyperoxaluria, low diuresis, intermittent moderate hyperoxaluria, Randall’s plaque
Ib	Whewellite	Stasis, low diuresis, crystalline conversion from weddellite to whewellite
Ic	Whewellite	Primary hyperoxalurias (mainly AGXT type 1 mutation)
Id	Whewellite	Malformative uropathy, stasis and confined multiple stones
Ie	Whewellite	Enteric hyperoxaluria, inflammatory (Crohn disease), ileal resections, chronic pancreatitis
IIa	Weddellite	Hypercalciuria with high calcium/citrate ratio
IIb	Weddellite	Hypercalciuria ± hyperoxaluria ± hypocitraturia, stasis, low diuresis
IIc	Weddellite	Hypercalciuria + malformative uropathy + stasis and confined multiples stones
IIIa	Uric acids anhydrous	Low urine pH, intermittent high uric acid, urine stasis, prostate hypertrophy
IIIb	Uric acid dihydrate ± uric acid anhydrous Urate salts	Low urine pH (metabolic syndrome, type 2 diabetes mellitus), high urinary uric acid, ammoniogenesis defect, hyperuricemia, myelo- and lymphoproliferative disorders
IIIc	Ammonium hydrogen urate	High urinary urate, alkaline urine pH
IIId	Ammonium hydrogen urate	High urinary urate, alkaline urine pH, malnutrition, low phosphate intake, excessive ammoniagenesis (infectieuse-urinary tract infection by urea-splitting micro-organisms or nutritional) Chronic diarrhea, electrolytes and alkali loss, low phosphate intake, laxative abuse, anorexia
IVa1	Carbapatite	Hypercalciuria, urinary tract infection see carbonatation rate of carbonated calcium phosphate
IVa2	Carbapatite	Inherited or acquired distal renal tubular acidosis, Sjogren syndrome, medullary sponge kidney
IVb	Carbapatite + other calcium phosphates (± struvite)	Urinary tract infection, hypercalciuria, primary hyperparathyroidism
IVc	Struvite	Urinary tract infection by urea-splitting bacteria
IVd	Brushite	Hypercalciuria, primary hyperparathyroidism, phosphate leak,
Va Vb	Cystine Cystine	Cystinuria Cystinuria + inadequate diet and/or medical management + stasis
VIa VIb VIc	Proteins Proteins + drugs or metabolic compounds Proteins + whewellite	Urinary tract infection, chronic pyelonephritis Example of drug-induced stone (mixture of proteins and atanazavir) End stage renal failure + relatively high urinary calcium concentration (long term calcium and Vitamin D therapy)
VII	Miscellaneous	
